# Fear-of-falling and associated risk factors in persons with rheumatoid arthritis: a 1 year prospective study

**DOI:** 10.1186/s12891-021-04068-0

**Published:** 2021-03-10

**Authors:** Emma K. Stanmore, Jackie Oldham, Dawn A. Skelton, Terence O’Neill, Mark Pilling, Chris Todd

**Affiliations:** 1 School of Health Sciences and Manchester Academic Health Science Centre (MAHSC), Jean McFarlane Building, University Place, University of Manchester, Manchester, M13 9LP UK; 2 Manchester University NHS Foundation Trust, M13 9WL Manchester, UK; 3 School of Health Sciences, Citylabs, Nelson Street, University of Manchester, Manchester, M13 9LP UK; 4 School of Health and Life Sciences, Glasgow Caledonian University, Cowcaddens Rd, Glasgow, G4 0BA UK; 5 Versus Arthritis Centre for Epidemiology and Centre for Musculoskeletal Research, University of Manchester, Manchester, M13 9PT UK; 6 Department of Rheumatology, Salford Royal National Health Service Foundation Trust, Salford, M6 8HD UK; 7 Behaviour and Health Research Unit, Forvie Site, University of Cambridge School of Clinical Medicine, Box 113 Cambridge Biomedical Campus, Cambridge, CB2 0SR UK

**Keywords:** Fear-of-falling, Falls, Physical function, Rheumatoid arthritis, Prospective

## Abstract

**Background:**

Falls, associated injuries and fear-of-falling are common in adults with RA. Fear-of-falling can be a major consequence of, and as debilitating as falling, resulting in a cycle of activity restriction, reduced quality of life, institutionalisation and potentially increase risk of falls. The objective of this study was to examine the relationship between fear-of-falling and risk factors associated with fear-of-falling in adults with rheumatoid arthritis (RA) over a 1 year period.

**Methods:**

Five hundred fifty-nine patients with RA were recruited from four outpatient clinics in this prospective cohort study. Baseline assessments included socio-demographic, medical and lifestyle related risk factors. Fall incidence was prospectively obtained monthly using postal cards over a 1 year period. Fear-of-falling was assessed at baseline and 1 year using the Short Falls Efficacy Scale-International (Short FES-I). Logistic regression was used to determine the association between high fear-of-falling (Short FES-I > 11) at baseline (outcome) and a range of putative predictor variables including previous falls, and also baseline factors associated with a high fear-of-falling at follow-up.

**Results:**

Five hundred thirty-five (ninety-six percent) participants (mean age 62.1 yrs.; 18–88 yrs) completed 1 year follow-up and of these, 254 (47%) completed the Short FES-I questionnaire at 1 year. In a multivariate model, a history of multiple falls (OR = 6.08) higher HAQ score (OR = 4.87) and increased time to complete the Chair Stand Test (OR = 1.11) were found to be independent predictors of high fear-of-falling and had an overall classification rate of 87.7%. There were no significant differences found in fear-of-falling at 1 year follow-up in those who reported falls during the study, participant’s baseline fear appeared to predict future fear, regardless of further falls.

**Conclusions:**

Fear-of-falling is significantly associated with previous falls and predictive of future falls and fear. RA patients would benefit from fall prevention measures whether or not they have previously fallen.

## Introduction

Patients with rheumatoid arthritis (RA) are at high risk of falls [1–3] and their associated injuries, including fractures and adverse psychological effects [1, 4, 5]. Given the adverse consequences of falls, it is not surprising that many people have a fear-of-falling. Such fear-of-falling can be as debilitating as falling itself and can result in activity restriction, reduced quality of life, increased use of medication, institutionalisation and may also increase the risk of injurious falls [6–10]. Fear-of-falling is not exclusively determined by physical weakness; many people with poor balance or a history of previous falls remain confident and do not report a fear-of-falling [11]. Although having a fall is linked with the development of fear-of-falling, fear-of-falling is not uncommon among those who have never fallen [12–14]. Falls and fear-of-falling are not limited to older people and research suggests they may affect younger as well as older adults with RA [15, 16]. Despite this, there are few data concerning the occurrence and determinants of fear-of-falling among people with RA [3]. Prevalence of fear-of-falling in cross-sectional RA studies shows marked variability (range:16–67%). This may in part be related to the study population (women only, small samples or frail older patients), inconsistent definitions of fear-of-falling and use of different assessment measures [5, 16–20]. To date, there are no prospective data concerning fear-of-falling or factors which predict fear-of-falling in adults with RA. The aim of this analysis was to determine factors linked with high fear-of-falling in men and women with RA, and factors linked also with the development of fear-of-falling.

## Methods

### Participants

Patients with a diagnosis of RA, based on the 2010 American College of Rheumatology classification criteria for RA [21] were consecutively recruited from four rheumatology clinics in the Northwest of England. Participants were excluded from the study if they were under the age of 18 years or if they were unable to give informed consent, as assessed by a trained research nurse. Data collection, analysis and reporting were completed between August 2008 and September 2013 and ethical approval for the study was obtained from the National Research Ethics Committee, reference 08/H1009/41 and written informed consent was obtained in accordance with the Declaration of Helsinki [22].

### Baseline assessment

Participants were assessed at baseline by a trained research nurse. This included an interviewer assisted questionnaire that included socio-demographic questions (gender, age, ethnicity, employment status, marital status), information about falls and fear-of-falling, and the underlying RA. A number of performance measures were also performed (see below).

i). Fear-of-falling, falls

At baseline the seven-item Short FES-I [23] was completed to measure confidence in performing a range of activities of daily living without falling. Activities include getting dressed or undressed, taking a bath or shower, getting in or out of a chair, going up or down stairs, reaching, walking up or down a slope, going out to a social event. A score is obtained by adding all the scores on all items together to give a total that ranges from 7 (no fear) to 28 (severe concern) [23]. Cut off points have been adopted to differentiate between lower and higher levels of fear-of-falling concern (7–10 = low concern, 11–28 = high concern) [11]. Participants were asked at baseline about their 1 year history of falls. A fall was classified using the Prevention of Falls Network Europe (ProFaNE) definition of, “an unexpected event in which participants come to rest on the ground, floor, or other lower level” [24].

ii). Medical and functional assessments

RA status was assessed using the Disease Activity Score (DAS28) [25]. Self-reported functional status was measured using the Health Assessment Questionnaire (HAQ) that measures patients’ perceptions of difficulties in performing activities in daily living, the need for equipment and physical assistance to perform tasks [26]. The validated Falls Risk Assessment Tool (FRAT) was used to measure falls risk [27]. Patients were also asked questions about levels of pain and fatigue using visual analogue scales (VAS) [28, 29], vision [30] and about any previous fractures, surgery, co-morbidities, or joint replacement(s) [31] and all verified using medical records. Medical records were also used to check medication, including steroid use and previous medical history [32]. Lower limb muscle strength was assessed using the Chair Stand Test [33] and balance was measured using the Four-Test Balance Scale [34].

### Follow up

Participants were asked to complete, over a course of a year, monthly follow-up of falls using prepaid, preaddressed calendar postcards that were filled in daily [35]. After 1 year participants were sent a postal questionnaire that included the Short FES-I [23].

### Sample size

The sample size calculation was based on fall rates as this was the primary focus of the study [1]. Based on data from retrospective studies [16, 31, 36] it was calculated that to estimate a fall rate of 0.3 falls/person year. a sample size of 495 (i.e. 550 people before an assumed 10% drop-out rate) would be able to estimate this to a precision of +/− 0.04 falls/person year (Clopper-Pearson [37], using StatsDirect version 3.1.14 [38]).

### Analysis

Statistical analysis was conducted using SPSS [39] and R version 3.4.3 [40]. Independent group t-tests for normally distributed data, or the equivalent non-parametric tests were used when appropriate to examine differences between those with low and high fear-of-falling. Paired t-tests or the equivalent non-parametric tests were used to examine differences between baseline and 1 year follow up. Chi-squared tests (or Fisher’s Exact test) were used to examine the differences in categorical variables between independent groups. Similarly ANOVA or the equivalent non-parametric test were used to compare between multiple independent groups. Bonferroni adjustments for multiple testing are considered in the interpretation of *p*-values (Table 2). The Short FES-I data (range: 7–28) were transformed into a binary variable indicating low (7–10) and high (11–28) fear-of-falling [11]. Checks were made to ensure that the sample remaining in the follow-up were not systematically different from non-responders. Binary logistic regression was used to calculate odds ratios (OR) and 95% confidence intervals (CI) for age and gender and all fear-of-falling risk associated variables including salient interaction terms. Model diagnostics (Hosmer-Lemeshow goodness-of-fit test, Q-Q plot for outliers) were checked and were satisfied [41]. Model building was conducted by first examining effect sizes in univariate logistic models using clinically relevant variables, and testing for multi-collinearity (variance inflation factors) in multivariate models. These model-building checks were satisfied.

## Results

### Participants

**Fig. 1:**
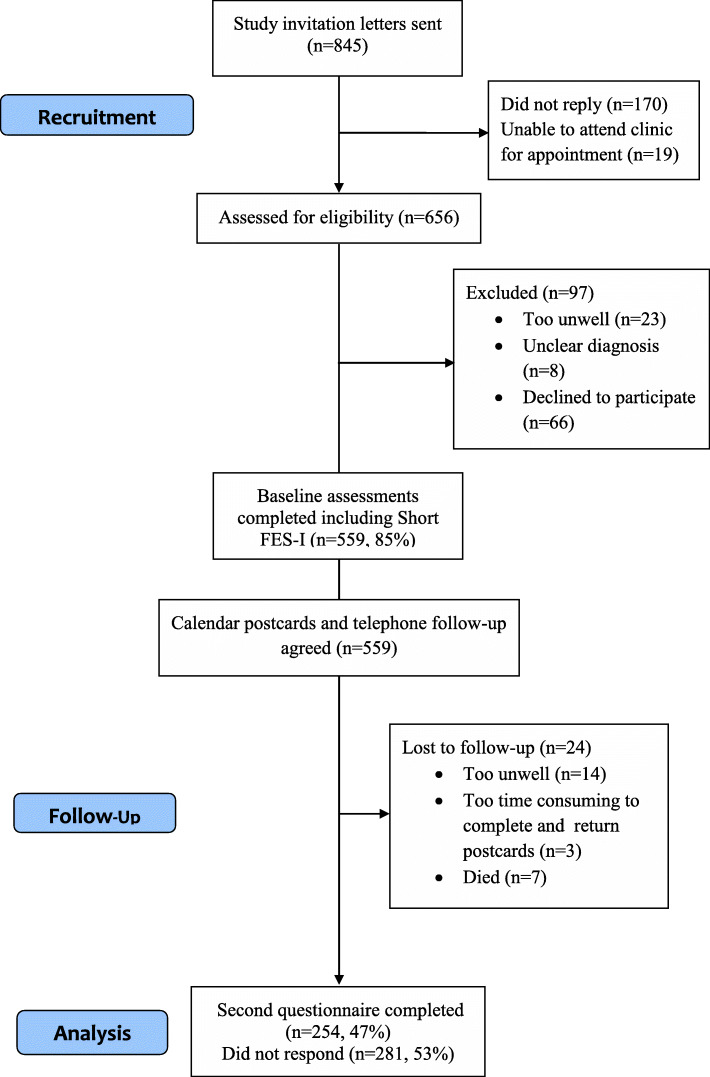
Flow diagram of study recruitment and follow up

A total of 559 participants completed the baseline assessments (Fig. 1) and 535 (96%) completed follow up of falls and remained in the study at 1 year (incidence of falls data reported elsewhere) [1]. Forty-seven percent (*n* = 254) of the participants completed the second postal Short FES-I questionnaire after 1 year. The mean age of participants was 62.1 years (SD = 12.8, median = 63; range 18–88) and 386 (69.1%) were women. The majority of participants were married or living with a partner (*n* = 378, 69.7%) and were of white British ethnicity and born in the UK (*n* = 528, 94.5%). Over half of the participants were retired (*n* = 327, 58.5%), and 14.7% were unable to work due to their disabilities (*n* = 82). About a quarter of participants (24%; *n* = 134) continued to be employed. The demographic, disease characteristics and fall risk factor variables of the study population at baseline (*n* = 559) and those continuing to the 1 year follow up (*n* = 254) are shown in Table 1 and demonstrate similar characteristics. The characteristics of those participants who did not complete the follow up questionnaire (*n* = 281, 53%) were also similar to those who did complete.

**Table 1:** Participant characteristics at baseline and 1 year follow up

Variable	Baseline (*n* = 559)	1 year follow up (*n* = 254)
**Demographics**		
Female n (%)	386 (69.1)	174 (68.5)
Age^a^	62 (12.8)	62 (10.0)
White British n (%)	534 (95.5)	240 (94.5)
Married/living with partner n (%)	378 (69.7)	199 (80.6)
Employed n (%)	134 (24.0)	55 (21.7)
**Disease factors**		
Swollen joints (0–28)^a^	4.6 (6.2)	4.3 (6.0)
Tender joints (0–28)^a^	5.3 (6.9)	5.4 (7.0)
DAS28 score (0–10)^a^	4.1 (1.6)	4.1 (2.6)
Taking steroids at baseline n (%)	117 (20.9)	47 (18.5)
VAS pain score (0–10)^a^	3.9 (2.6)	3.8 (2.6)
VAS fatigue score (0–10)^a^	4.7 (2.8)	4.5 (2.8)
HAQ score (1–4)^a^	2.4 (0.9)	2.4 (0.8)
**Medical history**		
History of fractures n (%)	228 (40.8)	107 (42.1)
Poor vision (reg. Blind, v poor or poor) n (%)	46 (8.2)	19 (7.5)
Number of co-morbidities ^a^	2.0 (2.0)	1.9 (1.9)
Previous surgery n (%)	408 (73.0)	187 (73.6)
Previous joint replacements^a^	0.2 (0.5)	0.2 (0.5)
Injuries from previous falls ^a^ n (%)	1.6 (1.4)	1.6 (1.4)
**Fall risk factors**		
History of stroke/Parkinsons n (%)	38 (6.8)	20 (7.9)
Psychotropic medication n (%)	104 (18.6)	51 (20.1)
Four or more types of medicines n (%)	431 (77.1)	196 (77.2)
Feeling dizzy or unsteady n (%)	370 (66.2)	167 (65.7)
Painful feet n (%)	431 (77.2)	192 (75.5)
Short FES-I score (7–28)^a^	15.3 (6.5)	14.8 (6.3)
Four test balance scale		
Fail at each level		
Unsuccessful n (%)	39 (7.0)	13 (5.1)
Feet together stand n (%)	13 (2.3)	7 (2.8)
Semi-tandem stand n (%)	216 (38.6)	93 (36.6)
Tandem stand n (%)	116 (20.8)	55 (21.7)
One leg stand n (%)	175 (31.3)	86 (33.9)
Able to complete 5 chair stands n (%)	484 (86.6)	217 (85.4)
Time taken for 5 chair stands (Seconds) ^a^	21.0 (12.2)	21.1 (13.2)

^a^mean (SD)

### Occurrence of falls

Detailed results on the incidence, risk factors and consequences of the falls are reported elsewhere [1, 15].

### Prevalence of fear-of-falling

At baseline, 69.9% of participants scored in the high fear-of-falling range (95% CI 66.1, 73.7%). At follow up 71.7% participants scored in the high fear-of-falling range (95% CI 65.4, 76.5%).

### Determinants of fear-of-falling

i). Retrospective data

Those who reported a fall in the previous year (*n* = 242) had a significantly higher Short FES-I score at baseline than those who reported no previous falls (*n* = 17.4 vs 13.8; *p* < 0.001). An independent t-test was used to compare the 1 year history of falls and the 1 year follow up Short FES-I score. There were no significant differences between the groups in mean follow up Short FES-I scores (t = − 1.5, df = 480, *p* = 0.1, 95% CI -2.6, 0.4).

Within the items of Short FES-I, getting dressed/undressed and getting into-out of a chair were of much less concern than the other items to the participants at baseline (*p* < 0.001).

Women (mean Short FES-I score = 16.0) had significantly higher fear-of-falling scores than men (mean Short FES-I score = 16.0 vs 13.9 respectively; t = − 3.7, df = 532, *p* < 0.001, 95% CI –3.23, − 1.01). Concerns about fear-of-falling consistently increased with age (in bands of up to 34, 35 to 44, 45 to 54, 65 to 74 and over 75 years; χ^2^ = 12.6, df = 1, *p* < 0.001).

#### Disease related factors

When compared with participants who had low fear-of-falling, participants who reported high concerns about fear-of-falling generally had more swollen/tender joints, higher DAS28, pain, fatigue and HAQ scores indicating more active RA disease and poorer physical function (all *p* ≤ 0.002) (Table 2). They also had more co-morbid conditions and had poorer lower limb strength as evidenced by more time needed to complete the five Chair Stand test.

**Table 2:** Fear-of-falling at baseline and 1 year follow-up

Variable	Baseline high fear-of-falling (***n*** = 390)^**b**^	Baseline low fear of falling (***n*** = 168)	Follow up high fear-of-falling (***n*** = 181)	Follow up low fear-of-falling (***n*** = 73)
**Demographics**				
Female (n/%)	287 (73.6)**	99 (58.9)	*122 (67.4)*	*52 (71.2)*
Age^a^	63 (12.7)**	59.9 (12.7)	62 (10.7)**	62 (10.0)
**Disease factors**				
Swollen joints (0–28) ^a^	5 (6.7)**	3 (4.7)	5.0 (6.4)**	2.6 (4.4)
Tender joints (0–28) ^a^	6 (7.3)**	3 (4.9)	6 (7.2)**	4.1 (6.3)
DAS28 score (0–10) ^a^	5 (1.5)**	3 (1.4)	5 (1.5)**	3 (1.6)
Taking steroids at baseline (n/%)	94 (24.1)*	23 (13.7)	42 (23.2)**	5 (6.8)
VAS pain score (0–10)^a^	5 (2.6)**	3 (2.2)	4 (2.5)**	3 (2.3)
VAS fatigue score (0–10)^a^	5 (2.6)**	3 (2.6)	5 (2.6)**	3 (2.6)
HAQ score (1–4) ^a^	3 (0.7)**	2 (0.5)	3 (0.8)**	2 (0.6)
**Medical history**				
History of fractures (n/%)	162 (41.5)	65 (38.7)	82 (45.3)	25 (34.2)
Poor vision (reg. Blind, v poor or poor) (n/%)	37 (9.5)	9 (5.4)	13 (7.2)	6 (8.2)
Previous surgery (n/%)	291 (74.6)	116 (69.0)	137 (75.7)	50 (68.5)
Previous joint replacements (n/%)	99 (25.6)	26 (15.5)	44 (24.6)	1 5 (15.1)
History of injuries from previous falls (n/%)	107 (30.7)**	17 (11.6)	48 (29.8)*	8 (12.1)
**Fall risk factors**				
No falls in previous year (n/%)	190 (48.7)**	126 (75.0)	91 (50.3)**	54 (74.0)
History of single fall in previous year (n/%)	87 (22.3)	33 (19.6)	42 (23.2)	13 (17.8)
History of multiple falls in previous year (n/%)	113 (29.0)	9 (5.4)	48 (26.5)	6 (8.2)
History of stroke/Parkinson’s (n/%)	31 (7.9)	7 (4.2)	18 (9.9)	2 (2.7)
Psychotropic medication (n/%)	87 (22.3)**	17 (10.1)	40 (22.1)	11 (15.1)
Four or more types of medicines (n/%)	328 (84.1)**	103 (61.3)	148 (81.8)*	48 (65.8)
Feeling dizzy or unsteady (n/%)	312 (80.0)**	57 (33.9)	136 (75.1)**	31 (42.5)
Painful feet (n/%)	321 (82.5)**	109 (64.9)	143 (79.4)	48 (65.8)
Four test balance scale (n/%)				
Fail at each level				
Unsuccessful	39 (10.0)**	0 (0.0)	13 (7.2)**	0 (0.0)
Feet together stand	13 (3.3)	0 (0.0)*	6 (3.3)	1 (1.4)
Semi-tandem stand	177 (45.4)	39 (23.2)*	75 (41.4)	18 (24.7)
Tandem stand	81 (20.8)	34 (20.2)*	41 (22.7)	14 (19.2)
One leg stand	80 (20.5)	95 (56.6)*	46 (25.4)	40 (54.8)
Able to complete 5 chair stands (n/%)	316 (81.0)**	167 (99.4)	145 (80.1)**	72 (98.6)

Bonferroni adjustment for multiple testing suggests *p* < 5%/25 = 0.002 for significance in comparisons at baseline, or comparisons at follow up

*P ≤ 0.01

**P ≤ 0.002

^a^Mean (SD)

^b^Data missing for 1 participant

At baseline, participants reporting high fear-of-falling concerns were more likely to be taking psychotropic medication, four or more medicines and steroids. High fear-of-falling levels were also more common in women and high fear-of-falling participants were associated with having a history of one or more falls, a history of stroke or Parkinson’s disease and a history of previous surgery and joint replacements. High levels of fear were also more common in participants who complained of feeling dizzy or unsteady, having painful feet, poorer balance and lower limb strength as tested by the four test balance scale and the ability to perform five chair stands (Table 2).

ii). Prospective data

Those who reported a fall during the study had a significantly higher mean baseline Short FES-I score of 16.8 than non- fallers at 14.4 (t = − 4.3, *p* < 0.001, 95% CI -3.5, − 1.3).

To examine whether fear-of-falling increased following a fall, an independent t-test was used on the change in Short FES-I score (the difference between follow-up Short FES-I score and baseline Short FES-I score). There were no significant differences in fear-of-falling found between non-fallers and fallers at baseline or follow-up (t = 0.8, df = 249, *p* = 0.41). Therefore, having a fall or not in the 1 year follow up did not appear significantly to change levels of fear-of-falling in this group of participants.Short FES-I scores did not differ between women and men at 1 year follow up (mean Short FES-I score = 15.3 vs 15.9 respectively; t = 0.8, df = 252, *p* = 0.41, 95% CI -0.9, 2.3). There were also no significant differences in levels of fear-of-falling with age (in bands of up to 34, 35 to 44, 45 to 54, 65 to 74 and over 75 year;χ^2^ = 0.07, df = 1, *p* = 0.8).

### Predictors of fear-of-falling

Following multivariable binary logistic regression, statistically significant variables were: a history of multiple falls (OR = 5.4, 95% CI 1.8 to 16.3, *p* = 0.02); painful feet (OR = 2.3, 95% CI 1.1 to 4.52, *p* = 0.03), increased HAQ score (OR = 7.8, 95% CI 3.9 to 15.6, *p* < 0.001), feeling dizzy or unsteady (OR = 3.8, 95% CI 2.0 to 7.2, p < 0.001) and increased time to complete the Chair Stand Test (OR = 1.1, 95% CI 1.0 to 1.1, *p* = 0.048). There was an inverse association between high fear-of-falling and swollen joints (OR = 0.9, 95% CI 0.85 to 0.99, *p* = 0.02) (Table 3). The fit of the predicted values in this logistic regression model was 85.5% and these results were also supported by a sensitivity analyses using bootstrapping (1000).

**Table 3:** Associations between risk factors and baseline fear-of-falling using multivariable binary logistic regression (high fear, *n* = 390/low fear, *n* = 168)

Risk factor	Score	Odds ratio (OR)	OR 95% Confidence Intervals	***p***-value
Gender	Male (referent) Female	1.24	0.64,2.37	0.65
Age	18–88	0.97	0.95,1.00	0.1
Number of swollen joints	0–28	**0.91**	**0.85,0.99**	**0.02**
Number of tender joints	0–28	0.97	0.89,1.04	0.4
DAS28 Score	0.1–8.9	1.37	0.95,1.99	0.1
Psychotropic medicines	No (referent)	1.15	0.44,3.00	0.8
	Yes			
Four or more types of medicines	No (referent)	1.74	0.82,3.68	0.1
	Yes			
Taking steroids at baseline	No (referent)	1.33	0.58,3.03	0.5
	Yes			
History of stroke or Parkinson’s disease	No (referent)	0.53	0.14,1.92	0.3
	Yes			
VAS pain score	0–10	1.00	0.85,1.16	0.9
VAS fatigue score	0–10	1.03	0.90,1.17	0.7
History of falls in previous 12 months	0 fall (referent) 1 fall 2 or more falls	1.56 **5.43**	0.70,3.52 **1.81,16.27**	0.3 **0.003**
Number of joint replacement	0–6	0.87	0.57,1.33	0.5
History of previous injuries	No (referent)	1.11	0.48,2.57	0.8
	Yes			
History of previous surgery	No (referent)	0.66	0.33,1.30	0.2
	Yes			
Painful feet	No (referent)	**2.34**	**1.11,4.52**	**0.025**
	Yes			
HAQ score	1.00–4.00	**7.79**	**3.87,15.69**	**< 0.001**
Four test balance scale^a^:				
Semi- tandem stand	2	1.15	0.50,2.64	0.7
Tandem stand	3	0.71	0.30,1.67	0.4
One – leg stand	4 (referent)			
Complaints of feeling dizzy or unsteady	No (referent)	**3.81**	**2.01,7.22**	**< 0.001**
	Yes			
Time taken for chair stand test	4–104 s	**1.05**	**1.00, 1.10**	**0.048**

Hosmer-Lemeshow goodness of fit test.

x^2^ = 5.0, df = 8, *p* = 0.759. Nagelkerke R^2^ = 61.3%

^a^Unable to add ‘feet together stand’ category as too few participants

A history of multiple falls (OR = 6.08, 95% CI 1.19 to 30.99, *p* = 0.03), higher HAQ scores (OR = 4.87, 95% CI 1.59 to 14.92, *p* = 0.006) and increased time to complete the Chair Stand Test (OR = 1.11, 95% CI 1.01 to 1.20, *p* = 0.02) were found to be the most significant predictors for future fear-of-falling (Table 4). Previous fear predicts future fear and 1 year later fear appeared to be lower amongst women than men. This model had an overall rate of correct classification of 87.7%. A sensitivity analyses using bootstrapping also supported these conclusions from the multivariate logistic regression model.

**Table 4:** Associations between risk factors and 1 year fear-of-falling using multivariable binary logistic regression (high fear, *n* = 181/low fear, *n* = 73)

Risk factor	Score	Odds ratio (OR)	OR 95% Confidence Intervals	***p***-value
Gender	Male (referent)	0.21	0.07, 0.63	0.05
	Female			
Age	18–88	0.95	0.89,1.00	0.06
Number of swollen joints	0–28	0.99	0.86, 1.13	0.8
Number of tender joints	0–28	0.92	0.82,1.03	0.1
DAS28 Score	0.1–8.9	1.31	0.70,2.44	0.4
Psychotropic medicines	No (referent)	1.11	0.31,4.00	0.9
	Yes			
Four or more types of medicines	No (referent)	0.81	0.26,2.52	0.7
	Yes			
Taking steroids at baseline	No (referent)	3.60	0.82,15.74	0.09
	Yes			
History of stroke or Parkinson’s disease	No (referent)	1.30	0.16,10.47	0.8
	Yes			
VAS pain score	0–10	0.96	0.75,1.22	0.7
VAS fatigue score	0–10	1.19	0.96,1.46	0.1
History of falls in previous 12 months	0 fall (referent) 1 fall 2 or more falls	2.44 6.08	0.68,8.71 1.19,30.99	0.2 **0.03**
Number of joint replacements	0–6	0.83	0.23,3.04	0.8
History of injuries from previous falls	No (referent)	0.81	0.22,2.95	0.8
	Yes			
History of previous surgery	No (referent)	1.15	0.41,3.28	0.8
	Yes			
Painful feet	No (referent)	0.92	0.32,2.64	0.8
	Yes			
HAQ score	1.00–4.00	4.87	1.59,14.92	**0.006**
Four test balance scale^a^:				
Semi- tandem stand	2	0.81	0.24,2.69	0.7
Tandem stand	3	1.40	0.37,5.40	0.6
One – leg stand	4 (referent)			
Complaints of feeling dizzy or unsteady	No (referent)	1.67	0.65,4.27	0.29
	Yes			
Time taken for chair stand test	4–104 s	1.11	1.01, 1.20	**0.02**
Baseline fear-of-falling	Low (referent)	30.05	1.42,637.18	**0.03**
	High			

Hosmer-Lemeshow goodness of fit test

x^2^ = 6.9, df = 8, *p* = 0.545. Nagelkerke R^2^ = 58.6%

^a^Unable to add ‘feet together stand’ category as too few participants

## Discussion

This is the first study that has prospectively investigated fear-of-falling and predictors for fear-of-falling in a sample of adults with RA. This study demonstrates that fear-of-falling is an important problem for adults of all ages with RA with some 70% of the population experiencing high levels of fear-of-falling, not limited to older people. Mean fear-of-falling scores were in the high fearful category for participants at baseline and at 1 year follow up and the prevalence of fear-of-falling appeared to increase with age and to be higher in women. This is consistent with community dwelling studies [8] and fear-related avoidance of activities is known to be predictive of future falls, physical frailty and reduced muscle strength [12]. Fear-of-falling in people with RA may affect their ability to carry out activities of daily living, such as going out to do shopping or to participate in a social event and so have negative psychological as well as physical consequences [5].

The prevalence of fear-of-falling in this study is higher than previous cross-sectional studies in those with RA that measured having fear or not [5, 16, 43] but this likely reflects the difference in measurement (Short FES-I vs single item measure). Having a fall or not in the 1 year follow up did not appear significantly to change levels of fear-of-falling in this group of participants. Those who fell in the previous 1 year may already experience higher levels of fear-of-falling that remain constant whether they fell again or not in the following 1 year.

Having had at least one previous fall was found to be an independent risk factor for fear-of-falling and nearly tripled the odds of having fear-of-falling. Increasing HAQ scores (but not disease activity scores) and increasing time to complete the Chair Stand Test (indicating a decrease in physical functioning) are other important independent risk factors for fear-of-falling. These results are similar to other studies that have demonstrated an association between functional decline and fear-of-falling in patients with RA [5, 19, 42, 43] and may be helpful in designing a screening tool for modifiable risk factors for patients at risk of fear-of-falling. By asking questions in routine assessments and reviews about fear-of-falling, falls and their context, health and social care practitioners may identify patients who may be at risk of falling. If there is concern that a person is at risk of falling, they can be referred to, or advised to see, a healthcare professional particularly a geriatrician or physiotherapist or falls prevention service if available, to further assess their risk and treat modifiable risk factors. Thus a recommendation for clinicians from this study would be to routinely monitor fear-of-falling amongst patients using a validated tool such as the Short FES-I.

A systematic review and meta-analysis of 30 trials by Kumar and colleagues found that exercise interventions (aimed at reducing falls) that included gait, balance, co-ordination, strength, resistance and functional tasks are associated with a small to moderate reduction of fear-of-falling in community-dwelling older adults [44]. However, a high risk of bias was noted throughout the included trials and so these results should be interpreted with caution, especially as most studies were short term with little long term follow-up to examine if the effect was sustained. There is good evidence that the home-based Otago exercise programme (OEP) [45] and group-based Falls Management Exercise (FaME) [46] strength and balance programmes are effective in reducing falls if progressive, tailored and reach a minimum effective dose. However, no study to date has investigated the effectiveness of these programmes specifically with patients with RA. Therefore, well designed future studies are needed to investigate whether exercise interventions are effective in reducing fear-of-falling, and falls in adults with RA.

This study has a number of limitations, these include the falls recorded during the study rely on self-report, low follow up response rate for the Short- FES-I and the method of recruitment of the participants from rheumatology clinics that may have resulted in participants with more moderate to severe RA or more progressive disease than the general primary care population. A measure of physical activity may have given further insight into whether fear-of-falling affected levels of activity. The rheumatology clinics were all based in the Northwest of England and therefore, the results should be interpreted with caution as they may not be generalisable to other populations.

## Conclusions

Fear-of-falling is common in adults with RA and this may alter activity levels and social engagement and may affect the ability to function independently. Fear-of-falling is significantly associated with previous falls and markers of poor function and is predictive of future falls and future fear. RA patients who report high fear-of-falling may benefit from fall prevention measures whether or not they have previously fallen, and exercise interventions to reduce fear-of-falling should also be considered.

## Data Availability

The datasets analysed during the current study are available from the corresponding author on reasonable request.

## Abbreviations

CI: Confidence Interval
DAS28: Disease Activity Score 28 joints
FaME: Falls Management Exercise
FRAT: Falls Risk Assessment Tool
HAQ: Health Assessment Questionnaire
OEP: Otago exercise programme
ProFaNE: Prevention of Falls Network Europe
RA: Rheumatoid arthritis
Short FES-I: Short Falls Efficacy Scale-International
VAS: Visual Analogue Scale
